# Newborn Mid–Upper Arm Circumference Identifies Low–Birth Weight and Vulnerable Infants: A Secondary Analysis

**DOI:** 10.1093/cdn/nzac138

**Published:** 2022-09-12

**Authors:** D Taylor Hendrixson, Patrick N Lasowski, Aminata Shamit Koroma, Mark J Manary

**Affiliations:** Department of Pediatrics, University of Washington, Seattle, WA, USA; Ichan School of Medicine at Mount Sinai, New York, NY, USA; Ministry of Health and Sanitation, The Republic of Sierra Leone, Freetown, Sierra Leone; Department of Pediatrics, Washington University School of Medicine in St. Louis, St. Louis, MO, USA

**Keywords:** LBW, low birth weight, MUAC, mid–upper arm circumference, neonatal anthropometrics, neonatal mortality, WLZ, weight-for-length *z*-score

## Abstract

**Background:**

Low birth weight (LBW) infants are at increased risk of morbidity and mortality. Identification of LBW may not occur in settings where access to reliable scales is limited. Mid–upper arm circumference (MUAC) may be an accessible, low-cost measure to identify LBW and vulnerable infants.

**Objectives:**

We explored the validity of newborn MUAC in identifying LBW and vulnerable newborns in rural Sierra Leone.

**Methods:**

This study was a secondary analysis of infant data from a randomized controlled clinical trial of supplementary food and anti-infective therapies compared with standard care for undernourished pregnant women. Data for singleton liveborn infants with birth measurement and 6-mo survival data were included in this analysis. The primary outcome was validity of MUAC in identifying low–birth weight (LBW) neonates. Secondary outcomes included validity of MUAC and head circumference (HC) in identifying weight-for-length *z*-score (WLZ) <−2, length-for-age *z*-score (LAZ) <−2, neonatal mortality, and mortality within the first 6 mo of life.

**Results:**

The study population included 1167 infants, 229 (19.6%) with LBW. Birth MUAC (*r* = 0.817) and HC (*r* = 0.752) were highly correlated with birth weight. MUAC (AUC: 0.905; 95% CI: 0.884, 0.925) performed superiorly to HC (AUC: 0.88; 95% CI: 0.856, 0.904) in identifying LBW. The MUAC for identifying LBW was 9.6 cm (sensitivity: 0.86; specificity: 0.78). Neither MUAC nor HC reliably identified newborns with WLZ <−2 or LAZ <−2. MUAC ≤9.0 cm was the ideal cutoff for neonatal mortality (sensitivity: 53.3%; specificity: 89.7%; HR: 9.57; 95% CI: 1.86, 49.30). Birth anthropometrics did not reliably identify infants at risk of death in the first 6 mo of life.

**Conclusions:**

MUAC was used successfully to identify LBW infants and infants at risk of neonatal mortality in Sierra Leone. Further evidence is needed to support increased use of newborn MUAC measurement to identify LBW infants and infants at risk of neonatal mortality in community settings where scales are not available. Primary trial was registered at clinicaltrials.gov as NCT03079388.

**Lay Summary:**

Mid–upper arm circumference (MUAC) can be used to identify infants with low birth weight and infants at risk for neonatal mortality, with an MUAC ≤9.0 cm indicating the highest risk.

## Teaser

MUAC (mid–upper arm circumference) can be used to identify infants with low birth weight and infants at risk for neonatal mortality, with an MUAC ≤9.0 cm conferring the highest risk.

## Introduction

Despite reductions in infant mortality rates over the last 30 y, neonatal mortality, especially in Sub-Saharan Africa, remains unacceptably high (1), underscoring the need to better identify neonates at risk for death so that targeted interventions may be administered. Low–birth weight (LBW) infants, defined as weighing <2500 g, have a 20-fold increased risk of death compared with normal-weight infants (2). LBW was defined in 1976 by the 29th World Health Assembly and correlates roughly with the 10th percentile of birth weight (3). Later in life, LBW survivors are more likely to suffer from stunting (4), wasting (4), lower cognitive capacity (5), and diabetes and obesity (6). Although LBW is a widely used indicator of risk of adverse outcomes among infants, it is an imperfect measure, and additional modifiers of risk include gestational age and small size for gestational age (7). In areas of the world where resources are scarce and home births are common, access to reliable scales for measuring birth weight may be limited. Additionally, large studies have demonstrated that even in large hospitals scales are not always available to weigh newborns (8). A simple and accurate anthropometric measurement is needed that detects at-risk neonates and provides useful information for referrals to specialized services in resource-poor rural areas.

Mid–upper arm circumference (MUAC) is a routinely used measurement to assess nutritional status in children aged 6–59 mo, often by community health workers in regions where undernutrition is prevalent (9–11). Therefore, MUAC may be a useful metric to identify newborns at risk for wasting, growth faltering, and death. However, data are limited regarding the use of MUAC in neonates in rural Sub-Saharan Africa. For most of the studies investigating proxy measurements for LBW, including MUAC, data were collected in a hospital setting and identified varying values for MUAC cutoffs between 8.7 and 9.8 cm (12–25). The limited number of studies conducted outside of hospital settings highlights the need for high-quality data on the use of anthropometric measurements to identify vulnerable infants in the community.

In this study, we analyzed data from a birth cohort in rural Sierra Leone to evaluate the validity of MUAC in the identification of newborn nutritional status and infants at high risk for neonatal mortality and mortality during the first 6 mo of life.

## Methods

This study was a secondary analysis of data from a prospective, randomized, controlled clinical effectiveness trial in which we compared the impact of a package of nutritional and anti-inflammatory interventions with the standard of care in undernourished pregnant women in Sierra Leone. Full details of the study design and interventions administered have been described previously (26, 27). The primary outcome for this analysis was validity of MUAC in identifying LBW neonates. Secondary outcomes included validity of MUAC and head circumference (HC) in identifying WLZ <−2, LAZ <−2, neonatal mortality, and mortality within the first 6 mo of life.

A total of 1489 women were enrolled in the primary clinical trial, and pregnancy outcomes were obtained from 1417 women. Women were enrolled at an average of 24 wk gestation, as assessed by fundal height, and randomly assigned to the intervention package or standard of care. Standard of care included corn-soy flour and intermittent preventative treatment for malaria. The intervention included replacing the flour with ready-to-use supplementary food and added azithromycin as well as testing and treatment for vaginal dysbiosis.

Clinic staff and participants notified the study coordinator at the time of delivery. A birth measurement team was dispatched to measure infants within 48 h of delivery. Birth measurements were taken as soon as was feasible, with a goal of within 72 h after birth. Infant weight, length, HC, MUAC, and morbidity were assessed at birth, 6 wk, 3 mo, and 6 mo of life. Nude weight was obtained using a Seca 334 infant digital scale accurate to 5 g. Recumbent length was measured in triplicate using a Seca 417 rigid height board; if the measurements differed by >2 mm, a fourth measurement was taken, and the 3 closest measurements were recorded and averaged. HC and MUAC of the left arm were obtained using a standard insertion tape accurate to the nearest 0.1 cm. Subsequently, if an infant was identified as deceased, the age at time of death was recorded.

### Participants

Pregnant women with undernutrition defined by an MUAC ≤23 cm and a fundal height <35 cm as a proxy for length of gestation were enrolled from 43 government antenatal clinics in Pujehun and the Western Rural Area Districts of Sierra Leone (26, 27). Exclusion criteria were known gestational diabetes, hypertension, or severe anemia.

All singleton live births born to mothers enrolled in the described clinical trial with complete birth measurements and follow-up survival data to 6 mo of life were included in the current analysis.

### Statistical analyses

Data were collected directly on clinic cards and then double entered into a spreadsheet database (Microsoft Access) and cross-checked for discrepancies. All discrepancies were resolved by examination of the original data card. No imputations or estimations were performed for missing data. Data were then anonymized. Once the content of the database was determined, it was locked for analyses.

Descriptive statistics were used to characterize the study population. Anthropometric parameters were converted to *z*-scores using the 2006 WHO growth standards (28). WLZ cannot be calculated for infants with lengths <45 cm; therefore, incalculable WLZ was evaluated as an additional risk category. All available values were included in the analysis.

Pearson correlation coefficients and linear regression analyses were performed to evaluate the relation of birth weight to MUAC and HC. Nonparametric receiver operator characteristic (ROC) curves were used to calculate 95% CIs of AUCs, as this allowed for the assessment of the performance of MUAC and HC to identify LBW, wasted, stunted, and underweight infants over a range of possible values (7). The Youden *J* statistic (*J* = sensitivity + specificity − 1), which assumes false positives and false negatives to be equally undesirable, was calculated to evaluate effectiveness and identify the optimal anthropometric cutoff (29).

Time-to-event values for mortality stratified by the identified anthropometric cutoffs were analyzed using both the Kaplan–Meier method with log-rank test and Cox proportional hazards regression, with the latter adjusted for maternal intervention received in the primary clinical trial and infant sex.

Statistical analysis was performed using IBM SPSS Statistics version 25 and GraphPad Prism version 8.3.0.

### Ethical considerations

This study was conducted according to the guidelines laid down in the Declaration of Helsinki and all procedures involving research study participants were approved by the Sierra Leone Ethics and Scientific Review Committee (SLESRC) and from the Human Research Protection Office at Washington University in St. Louis(ID# 201611119). Informed consent for participation in the primary clinical trial and secondary use of data was obtained for eligible and interested women by a signature or thumbprint if the participant was unable to write. Participants received nutritional supplementation for the duration of pregnancy and incentives for postpartum followup visits. Participants directly benefited from the clinical trial in receiving nutritional supplementation and quality antenatal care. Results of the primary clinical trial were disseminated to local communities. The primary clinical trial was registered at clinicaltrials.gov (NCT03079388).

## Results

In total, 1167 infants were included in this analysis (**Supplemental Figure S1**). Birth measurements were obtained at a median of 1 d of life (IQR: 2 d). Among this cohort, 229 (19.6%) were LBW. Descriptive statistics of birth anthropometrics are presented in **Table 1**.

**TABLE 1:** Baseline cohort anthropometric and mortality characteristics.^1^

Characteristic	*n* = 1167
Female	586 (50.2)
BW	2.858 ± 0.454
LBW (<2.500 kg)	229 (19.6)
Very LBW (<1.500kg)	6 (0.5)
Extremely LBW (<1.000kg)	0
WAZ	−1.06 ± 1.00
WAZ −2 to −3	116 (9.9)
WAZ <−3	49 (4.2)
Length, cm	47.1 ± 2.4
LAZ	−1.5 ± 1.1
LAZ -2 to -3, *n*	274 (23.5)
LAZ <−3, *n*	100 (8.6)
MUAC, cm	9.8 ± 0.8
HC, cm	33.9 ± 1.5
HcAZ	−0.4 ± 1.2
HcAZ −2 to −3	82 (7.0)
HcAZ <−3, *n*	23 (2.0)
WLZ	0.05 ± 1.05
WLZ −2 to −3	26 (2.2)
WLZ <−3	9 (0.8)
Unable to calculate WLZ^2^	184 (15.8)
Total deaths	63 (5.4)
Neonatal deaths	15 (1.35)

1 Values expressed as mean ± SD or *n* (%). BW, birth weight; HC, head circumference: HcAZ, head circumference-for-age *z*-score; LAZ, length-for-age *z*-score; LBW, low birth weight; MUAC, mid–upper arm circumference; WAZ, weight-for-age *z*-score; WLZ, weight-for-length *z*-score.

2 Birth length <45 cm, therefore, incalculable WLZ.

**FIGURE 1:**
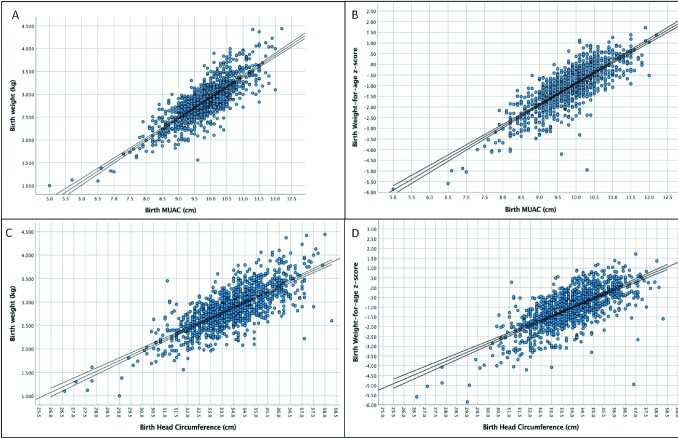
Scatterplots and linear regression lines for LBW and differing anthropometric indices. Scatterplot of birth weight (kg) and birth MUAC (cm) with linear regression line (A). Scatter plot of birth WAZ and birth MUAC (cm) with linear regression line (B). Scatter plot of birth weight (kg) and birth head circumference (cm) with linear regression line (C). Scatterplot of birth WAZ and birth head circumference (cm) with linear regression line. MUAC, mid–upper arm circumference; WAZ, weight-for-age *z*-score.

MUAC was strongly correlated with birth weight (*r* = 0.817, *P* < 0.001) (**Figure 1**A) and birth weight-for-age *z*-score (WAZ) (*r* = 0.804, *P* < 0.001) (Figure 1B). The predicted birth MUAC for LBW by linear regression equation was 9.3 cm. Birth HC was strongly correlated with birth weight (*r* = 0.752, *P* < 0.001) (Figure 1C) with a predicted birth HC for LBW by linear regression equation of 33.0 cm. Birth HC was less strongly correlated with birth WAZ (*r* = 0.695, *P* = <0.001) (Figure 1D).

**FIGURE 2:**
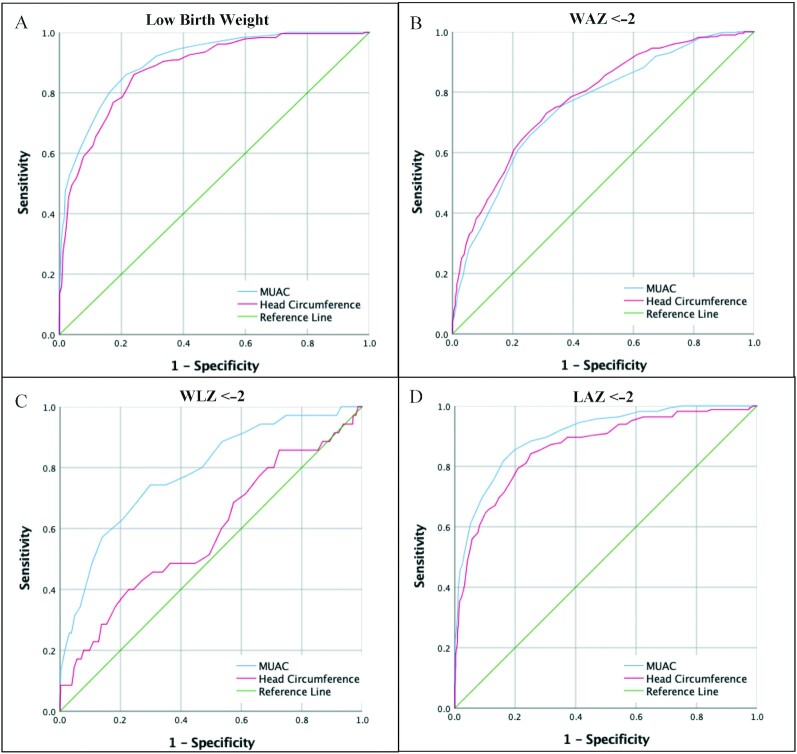
ROCs evaluating diagnostic validity for birth MUAC and HC for differing anthropometric indices. ROC evaluating diagnostic validity of birth MUAC (cm) and birth head circumference (cm) for low birth weight (A). ROC evaluating diagnostic validity of birth MUAC (cm) and birth head circumference (cm) for detecting infants with WAZ < −2 SD (B). ROC evaluating diagnostic validity of birth MUAC (cm) and birth head circumference (cm) for detecting infants with WLZ < −2.0 SD (C). ROC evaluating diagnostic validity of birth MUAC (cm) and birth head circumference (cm) for detecting infants with LAZ < −2.0 SD (D). LAZ, length-for-age *z*-score; MUAC, mid–upper arm circumference; WAZ, weight-for-age z-score; WLZ, weight-for-length *z*-score.

ROC curves were created to evaluate MUAC and HC for the detection of LBW, WAZ < −2, weight-for-length *z*-score (WLZ) < −2, and length-for-age (LAZ) < −2 at birth (**Figure 2**). MUAC was best for the detection of LBW (AUC: 0.91; 95% CI: 0.88, 0.93). The optimal MUAC cutoff for identifying LBW infants was 9.6 cm. HC performed well (AUC: 0.88; 95% CI: 0.86, 0.90) with an optimal cutoff of 33.5 cm. MUAC was best for detection of underweight (AUC: 0.90; 95% CI: 0.88, 0.93) (Figure 2B) with an optimal MUAC cutoff of 9.5 cm. HC also identified underweight infants well (AUC: 0.86; 95% CI: 0.83, 0.90) with a cutoff of 33.4 cm (Figure 2B). Neither MUAC nor HC performed as well at detecting infants with WLZ < −2 or LAZ < −2 (Figure 2C and D).

During the neonatal period, 15/1167 (1.3%; 95% CI: 0.8%, 2.1%) infants died. Neonatal deaths occurred at a median of 15 d (IQR: 18) with 3 deaths (20%) occurring in the first 3 d of life. Four (26.7%) of the infants that died had birth weights <1.500 kg. Birth MUAC demonstrated moderate diagnostic validity for identifying neonates at risk of neonatal mortality (AUC: 0.71; 95% CI: 0.53, 0.89). A birth MUAC ≤9.0 cm was identified as the optimal cutoff (sensitivity: 53.3%, specificity: 89.7%, Youden *J*: 0.43). Use of a MUAC cutoff of ≤9.6 cm demonstrated a sensitivity of 73.3%, a specificity of 66.3% and a Youden *J* of 0.40. Birth HC also demonstrated moderate diagnostic validity in identifying neonates at-risk for neonatal mortality (AUC: 0.76; 95% CI: 0.59, 0.93) with a cutoff of ≤32.2 cm (sensitivity: 66.7%, specificity: 89.4%, Youden *J*: 0.56). Using a birth HC of ≤33.5 cm, which identified LBW infants, resulted in a sensitivity of 73.3%, specificity of 64.3%, and a Youden *J* of 0.38.

**FIGURE 3:**
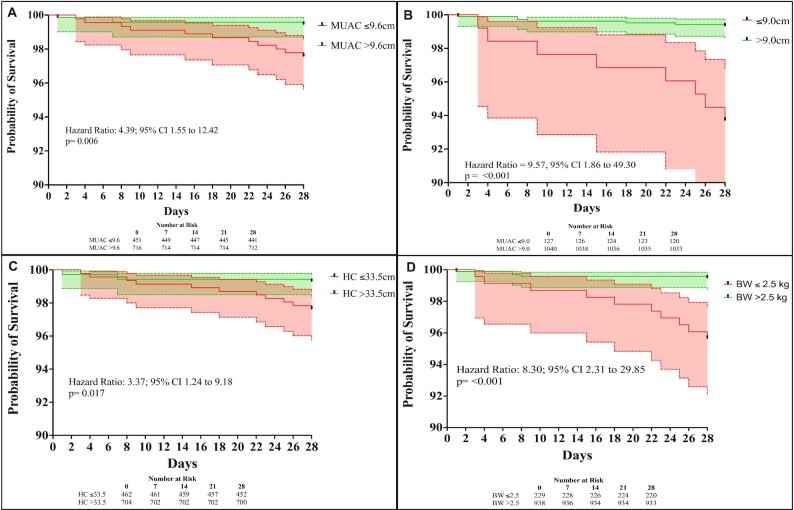
Kaplan–Meier survival curves for neonatal mortality according to varying anthropometric cutoffs. Birth MUAC ≤9.6 cm for neonatal survival (A); birth MUAC ≤9.0 cm for neonatal survival (B); birth HC ≤33.5 cm for neonatal survival (C); BW ≤2.5 kg for neonatal survival (D). BW, birth weight; HC, head circumference; MUAC, mid–upper arm circumference

Kaplan–Meier survival curves using MUAC and LBW were constructed to determine the risk of death in the neonatal period among the cohort. Infants with an MUAC of 9.6 cm, as this cutoff identified LBW infants, carried an HR for neonatal mortality of 4.39 (95% CI: 1.55, 12.42, *P* = 0.006) (**Figure 3**A, **Supplemental Table S1**). An MUAC of 9.0 cm was the best cutoff for neonatal mortality, resulting in an HR of 9.57 (95% CI: 1.86, 49.30, *P* < 0.001) (Figure 3B, Supplemental Table S1). Neonatal survival was decreased among infants with HC ≤33.5 cm, which was selected as the cutoff for LBW diagnosis in infants, compared with neonatal survival in infants with HC >33.5 cm (HR: 3.37; 95% CI: 1.24, 9.18; *P* = 0.017) (Figure 3C, Supplemental Table S1). Infants with birth HC ≤32.2 cm had an HR for neonatal mortality of 13.87 (95% CI: 3.02, 63.70, *P* = < 0.001) (Supplemental Table S1). Among LBW infants, the HR for neonatal mortality was 8.30 (95% CI: 2.31, 29.85, *P* = < 0.001) (Figure 3D, Supplemental Table S1).

During the 6-mo period, a total of 63/1167 (5.4%; 95% CI: 4.2%, 6.8%) died. Among infants born LBW, there were 18/229 (7.9%; 95% CI: 5.0%, 12.1%) deaths in the first 6 mo of life, accounting for 28.6% (95% CI: 18.9%, 40.7%) of deaths in the cohort. All birth anthropometrics demonstrated AUCs of >0.5 for identifying infants at risk of mortality in the first 6 mo of life; however, all performed relatively poorly (**Supplemental Table S2**, **Supplemental Figure S2**). Neither birth MUAC (cutoff: 9.9 cm; sensitivity: 50%; specificity: 57.6%; Youden *J*: 0.076) nor birth HC (cutoff: 35.4 cm; sensitivity: 91.3; specificity: 19.2%; Youden *J*: 0.105) demonstrated diagnostic validity in identifying infants at risk for mortality in the first 6 mo of life. Birth length demonstrated the best diagnostic validity for identifying infants at risk of mortality in the first 6 mo of life (AUC: 0.60; 95% CI: 0.52, 0.67) with a cutoff of 48.2 cm (sensitivity: 87%, specificity: 35.4%, Youden *J*: 0.22), followed by birth weight (AUC: 0.56; 95% CI: 0.49, 0.64) with a cutoff of 2.965 kg (sensitivity: 71.7%, specificity: 45.4%, Youden *J*: 0.17).

Time-to-event for mortality stratified by the identified MUAC thresholds and *z*-scores for other birth anthropometrics were analyzed using both the Kaplan–Meier method with a log-rank test and Cox proportional hazards to determine the hazard of death in the first 6 mo of life among infants in the cohort. Birth weight <2.0 kg carried the highest hazard of death at 6 mo of life (HR 6.6; 95% CI: 3.1, 14.6, *P* = < 0.001), followed by birth HC-for-age *z*-score <−3 (**Table 2**). Birth LAZ <−3, WLZ that could not be calculated, and MUAC ≤9.0 cm all carried elevated HRs for death by 6 mo of life (Table 2). HRs for mortality at 6 mo for all anthropometric measures were lower than those identified for neonatal mortality (Supplemental Table S1).

**TABLE 2:** Birth anthropometry and risk of death by 6 mo of life.^1^

Birth anthropometry	*N*	Death, *n* (%)	HR	95% CI	*P* value	AHR^1^	95% CI	*P* value
Weight, kg								
≥2.5 kg	950	45 (4.7)	Ref	Ref	Ref	Ref	Ref	Ref
2.0–2.4 kg	183	10 (5.5)	1.2	0.6–2.5	0.6	1.3	0.6–2.5	0.5
<2.0 kg	34	8 (23.5)	6.4	1.2–33.8	<0.001	6.6	3.1–14.6	<0.001
WAZ								
WAZ >−2	1002	48 (4.8)	Ref	Ref	Ref	Ref	Ref	Ref
WAZ −2 to −3	116	6 (5.2)	1.2	0.5–3.0	0.6	1.7	0.7–9.9	0.3
WAZ <−3	49	9 (18.4)	2.5	0.70–8.7	0.2	2.4	1.1–5.0	0.04
LAZ								
LAZ >−2	1002	48 (4.8)	Ref	Ref	Ref	Ref	Ref	Ref
LAZ −2 to −3	274	17 (6.2)	1.3	0.7–2.5	0.3	1.1	0.5–2.0	0.9
LAZ <−3	100	11 (11)	2.9	1.1–7.7	0.001	2.5	1.2–5.5	0.02
MUAC, cm								
>9.6	716	35 (4.9)	Ref	Ref	Ref	Ref	Ref	Ref
9.1–9.6	299	13 (4.3)	0.6	0.4–1.2	0.1	0.7	0.4–1.2	0.2
≤9.0	152	15 (9.9)	2.2	1.3–5.6	0.01	2.1	1.2–3.9	0.02
HcAZ								
HcAZ >−2	1062	50 (4.7)	Ref	Ref	Ref	Ref	Ref	Ref
HcAZ −2 to −3	82	6 (7.3)	2.03	0.7–5.9	0.2	1.3	0.6–3.2	0.5
HcAZ <−3	23	7 (30.4)	9.1	1.1–76.5	<0.0001	5.7	2.2–25.2	<0.001
WLZ								
WLZ >−2	948	45 (4.7)	Ref	Ref	Ref	Ref	Ref	Ref
WLZ −2 to −3	26	1 (3.8)	0.9	0.1–6.1	0.9	0.9	0.1–6.7	0.9
WLZ <−3	9	0	0.4	0.02–8.8	0.5	—	—	—
Unable to calculate WLZ^2^	184	17 (9.2)	2.2	1.1–4.6	0.003	2.3	1.3–4.2	0.004

1 Adjusted for maternal intervention received in primary trial and infant sex. HcAZ, head circumference for age *z*-score; LAZ, length for age *z*-score; MUAC, mid–upper arm circumference; Ref, reference; WAZ, weight-for-age *z*-score; WLZ, weight-for-length *z*-score.

2 184 infants had length <45 cm and therefore incalculable WLZ.

## Discussion

A low-cost, easily accessible community measurement for LBW infants in areas where access to perinatal healthcare is reduced and neonatal mortality is high could aide in the detection of LBW infants and facilitate appropriate referral and support. Previous studies seeking to find a surrogate for LBW considered 4 anthropometric measurements, neonatal foot length (16–19, 23, 30–34), chest circumference (12–14, 16, 17, 20, 21, 23, 25, 31, 33, 35, 36), MUAC (12–14, 16, 21–25, 33–37), and HC (13, 17, 20, 22, 25). However, the use of chest circumference and foot length measurements are not routine in most settings. Implementing a screening program based on one of these measurements would require an additional investment in training and equipment. There is no single gold-standard anthropometric measure of risk, and any measure should be selected based on consideration of its simplicity, acceptability, precision, accuracy, cost, and sensitivity/specificity (38, 39).

MUAC identified LBW neonates born to malnourished women in rural Sierra Leone. Neither MUAC nor HC performed reliably in identifying infants with WLZ <−2 or LAZ <−2. This finding is not surprising as neither WLZ nor LAZ has been shown to be a good predictor of risk among this age group. Birth MUAC and HC can also be used to identify infants at risk for neonatal mortality. Mortality cutoffs differed from those identified for LBW neonates. Using the MUAC and HC cutoffs that identified LBW neonates, an MUAC cutoff of ≤9.6 cm identifies infants at high risk of neonatal mortality, although the specificity is low. Neonates with an MUAC of ≤9.0 cm had a higher hazard of neonatal mortality than any of the birth anthropometrics, even birth weight. Additionally, an MUAC of ≤9.0 cm carried an elevated hazard for death by 6 mo of life. Use of a single MUAC cutoff of ≤9.0 cm may allow for ease of referral and adoption in limited resource settings and is consistent with findings in a previously published cohort in Burkina Faso (40). None of the birth anthropometrics performed optimally for identifying neonates at risk for mortality in the first 6 mo of life, though birth weight <2.0 kg carried the highest hazard of death.

MUAC has the greatest potential for utility in settings where scales are not available as it is easily and reliably performed. A key advantage of MUAC is that its use is already widespread in many countries as an indicator of nutritional status in children aged >6 mo. For example, the Ministry of Health and Sanitation of Sierra Leone collects information on nutritional status, including MUAC, via monthly surveys and mandates that community health workers (CHWs) and mother support groups be trained in its use to identify malnutrition in children (9). The type of tape and procedure used for measuring MUAC in a newborn is identical to what is already used in older children. An MUAC tape for use in infants <6 mo old has been developed by GOAL and is available online. Therefore, implementing an MUAC-based screening program for newborns would be straightforward, even in rural areas where CHWs and mother support groups are the only healthcare workers to which neonates consistently have access. That MUAC data can be accurately obtained by caregivers and CHWs when advanced healthcare providers are not available is an important advantage (41, 42).

Additionally, most studies investigating proxy measurements for LBW have collected data in hospital settings (12–25). Population characteristics of infants born in a hospital setting may not be equivalent to those of infants born in community settings (43). Proxy measurements for LBW are most likely to be useful in low-resource rural community health centers and for in-home births, where accurate scales are scarce. A study from rural Burkina Faso found a birth MUAC of 9.7 cm to have a sensitivity of 72.3% and specificity of 84.6% for identifying LBW in infants and that a birth MUAC <9.0 cm was associated with an elevated hazard of death (HR: 3.97) (40), very similar to the results of our study.

Strengths of this study include a large study population, standardized collection of all anthropometric measurements taken within 72 h of birth by a specifically trained technician, and the taking of all measurements in the participant's home community or peripheral health unit. Limitations of this study are that the study population was taken from undernourished pregnant women, which may not reflect the population of all pregnant women. The rate of LBW (14%) in our study is higher than that reported in Sierra Leone (∼6%) (44). An additional limitation was that foot length and chest circumference were not included in our screening of newborn infants. This limitation makes it difficult to compare our data on MUAC and HC with other data on chest circumference and foot length. We did not collect gestational age from ultrasound dating, so identifying the effect of small for gestational age or prematurity status on birth MUAC was not possible. The number of deaths among the cohort was low, and additional confounders may also have affected risk of mortality, including socioeconomic status, feeding practices, and other healthcare-seeking behaviors. We only assessed for mortality among the infants; however, as child survival increases assessment of risk for morbidities should be evaluated. Our findings are applicable to rural Sierra Leone and may not apply to other settings as infants in different geographic areas are known to have different average anthropometric birth measurements that are considered healthy and normal (35).

Given the effectiveness of MUAC as a screening tool for LBW and neonatal mortality in rural Sierra Leone and the existing widespread use of MUAC screening, future research should investigate the feasibility and effectiveness of using MUAC to identify LBW in other high-risk populations in resource constrained environments. MUAC can be quickly obtained, is simple to perform, and does not require calibration or sophisticated equipment. If data continue to show MUAC to be a sensitive and specific marker for LBW and vulnerable neonates, the introduction of large-scale screening programs for neonates in areas of the world where mortality is high could decrease the number of unrecognized and inadequately supported neonates, helping to achieve UN Sustainable Development Goal 3.2 (45).

## Supplementary Material

nzac138_Supplemental_File

## Data Availability

Data cannot be shared publicly because of the presence of possible participant identifiable content after de-identification.

## Notes

Study funding for the primary clinical trial was provided by the Children's Investment Fund Foundation. The study funders played no role in the design, conduct of the study, data analyses, and publication decisions.

The authors reported no funding received for this study.

Author disclosures: The authors report no conflicts of interest.

Supplemental Figures 1–2 and Supplemental Tables 1–2 are available from the “Supplementary data” link in the online posting of the article and from the same link in the online table of contents at https://academic.oup.com/cdn/.

Abbreviations used: HC, head circumference; LAZ, length-for-age *z*-score; LBW, low birth weight; MUAC, mid–upper arm circumference; ROC, receiver operator characteristic; WAZ, weight-for-age *z*-score; WLZ, weight-for-length *z*-score
