# Supplementary material for: Newborn Mid–Upper Arm Circumference Identifies Low–Birth Weight and Vulnerable Infants: A Secondary Analysis
Source: Curr Dev Nutr. 2022 Sep 12;6(10):nzac138. doi: 10.1093/cdn/nzac138 (PMC9718650; doi:10.1093/cdn/nzac138)
Supplement: nzac138_Supplemental_File [file nzac138_supplemental_file.pdf]

SUPPLEMENTARY MATERIALS

Newborn Mid-Upper Arm Circumference Identifies Low-Birth Weight and Vulnerable Infants: A Secondary Analysis

Hendrixson DT *et al*

Supplementary Table 1. Hazard ratios for neonatal mortality and 6-month mortality for MUAC, head circumference and LBW.

	**Neonatal Mortality**			**6-month Mortality**		
**Birth anthropometry**	**Hazard Ratio**	**95% CI**	**p-value**	**Hazard Ratio**	**95% CI**	**p-value**
MUAC						
>9.6 cm	Reference					
9.0-9.6 cm	4.39	1.55, 12.42	0.006	0.6	0.4 to 1.2	0.1
≤9.0 cm	9.57	1.86, 49.30	<0.001	2.2	1.3, 5.6	0.01
Head Circumference						
>33.5 cm	Reference					
≤33.5 cm	3.37	1.24, 9.18	0.017	1.16	0.70, 1.92	0.57
≤32.2 cm	13.87	3.02, 63.70	<0.001	2.05	1.08, 3.89	0.03
Low Birth Weight						
>2.5 kg	Reference					
≤2.5 kg	8.30	2.31, 29.85	<0.001	2.03	1.07, 3.86	0.03

Supplementary Table 2. Receiver operating characteristic curve AUC values for birth anthropometry to identify mortality at 6-months of age

Birth anthropometry	N	Deaths	AUC	95%CI
Birth Weight, kg	1167	63	0.562	0.486 to 0.639
Birth Length, cm	1167	63	0.599	0.523 to 0.674
MUAC, cm	1167	63	0.522	0.444 to 0.599
Head Circumference, cm	1167	63	0.512	0.434 to 0.590
WLZ	983	46	0.544	0.460 to 0.628

Abbreviations: AUC- area under the curve; 95% CI- 95% confidence interval; MUAC- mid-upper arm circumference; WLZ- weight-for-length z-score

[1]184 infants had length <45 cm and therefore incalculable WLZ.

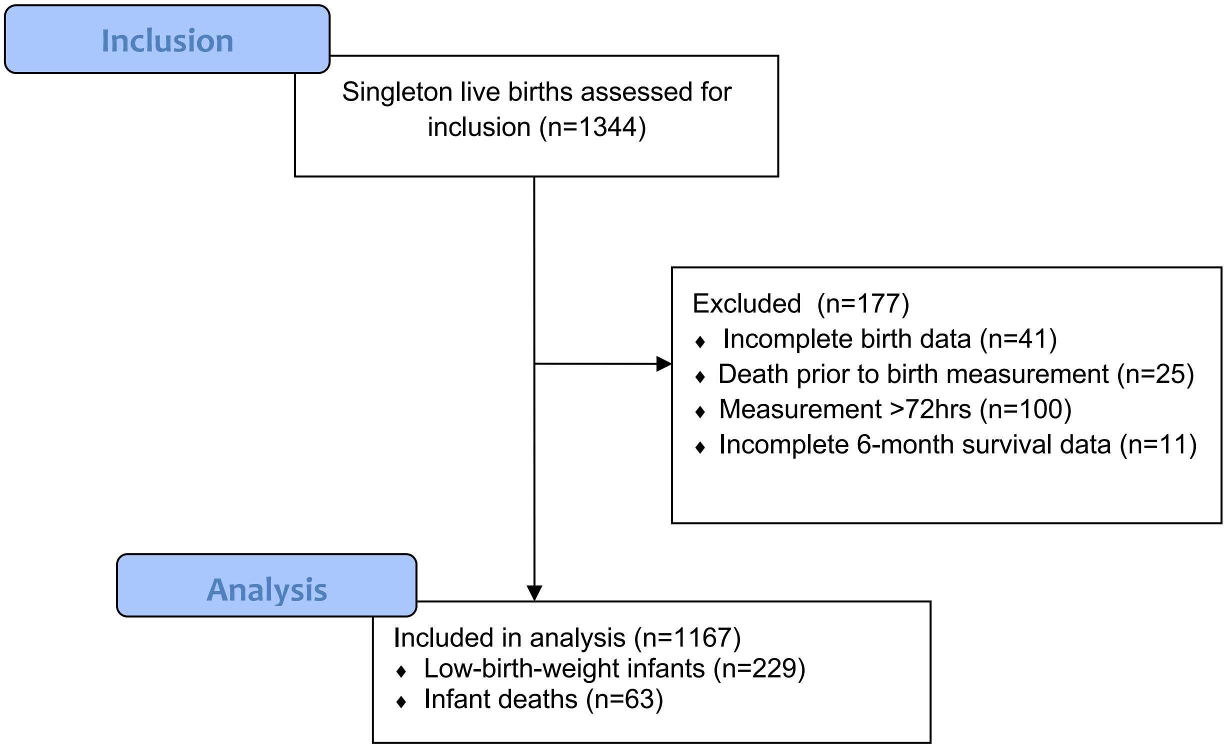

Supplemental Figure 1. Patient flow diagram

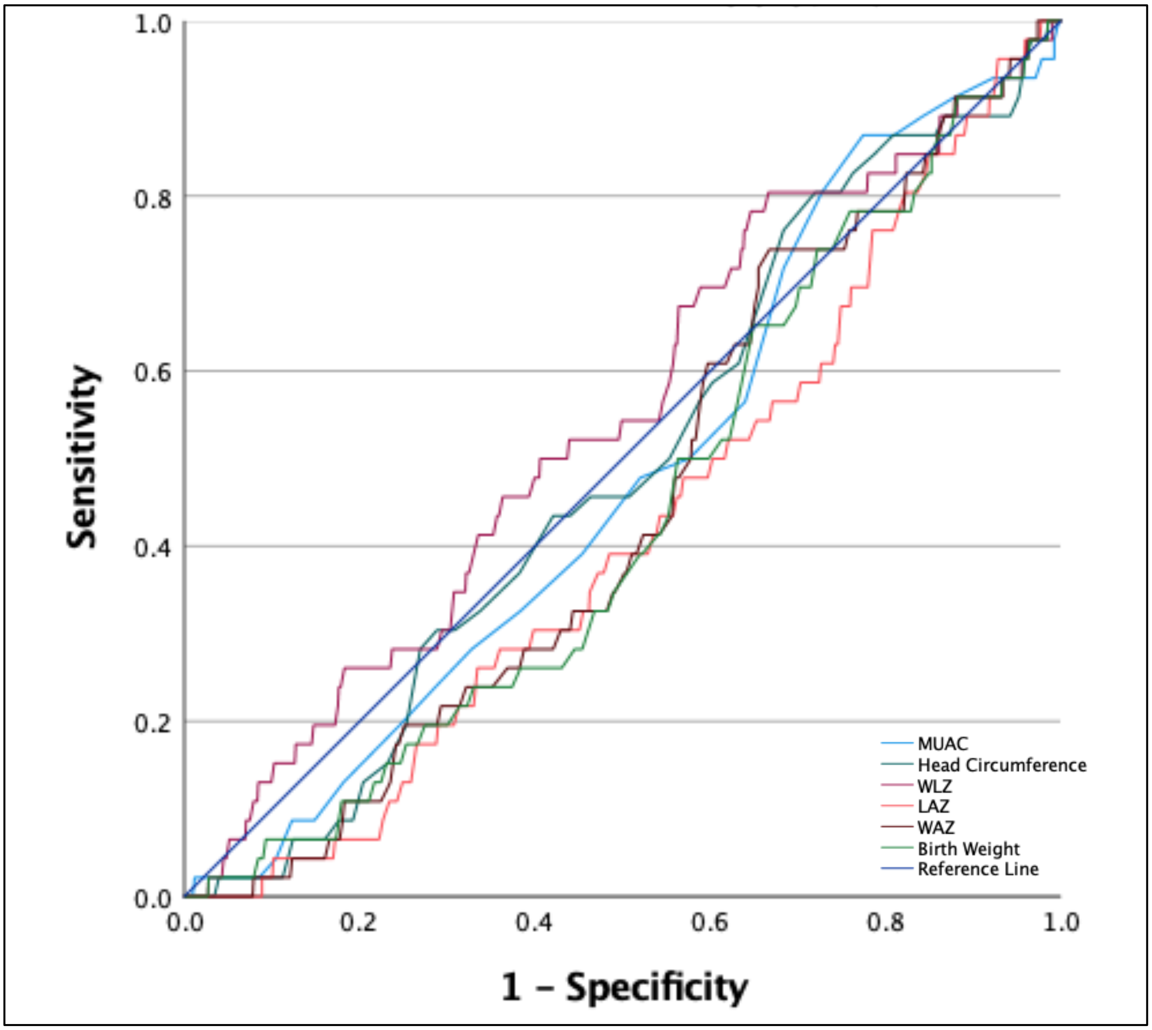

Supplementary Figure 2- Receiver operator curve evaluating diagnostic accuracy of birth MUAC (cm), head circumference (cm), weight (kg), LAZ, WAZ and WLZ for identifying infants at risk for mortality in the first 6 months of life.
